# A prospective cohort study of the SEARCH integrated HIV/hypertension community health worker‐led intervention in rural Kenya and Uganda

**DOI:** 10.1002/jia2.26500

**Published:** 2025-07-07

**Authors:** Matthew D. Hickey, Asiphas Owaraganise, Sabina Ogachi, Helen Sunday, Colette Aoko, Norton Sang, George Agengo, Jane Kabami, Elijah Kakande, Erick Wafula Mugoma, Josh Schwab, Nicole Sutter, Douglas Black, Anthony Muiru, Gabriel Chamie, Maya L. Petersen, Laura B. Balzer, Elizabeth A. Bukusi, Diane V. Havlir, Moses R. Kamya, James Ayieko

**Affiliations:** ^1^ Division of HIV, Infectious Disease, & Global Medicine University of California San Francisco California USA; ^2^ Infectious Diseases Research Collaboration Kampala Uganda; ^3^ Kenya Medical Research Institute Nairobi Kenya; ^4^ School of Public Health University of California Berkeley California USA; ^5^ Division of Nephrology University of California San Francisco California USA; ^6^ School of Medicine Makerere University Kampala Uganda

**Keywords:** community health worker, HIV, hypertension, integrated care, telehealth, telemedicine

## Abstract

**Introduction:**

Clinic‐based hypertension screening and treatment for people with and without HIV depends on consistent clinic engagement. Retention is challenging in rural areas, especially for people with severe hypertension, which typically requires more frequent visits than clinically stable HIV. We hypothesised that Ministry of Health (MoH) community health workers (CHWs) could improve severe hypertension detection and treatment through an integrated hypertension/HIV intervention.

**Methods:**

In rural Uganda and Kenya, we added HIV testing and a status‐neutral hypertension intervention to CHW workflow in an ongoing cluster‐randomised population‐level study (SEARCH:NCT05768763). Data spans March 2023–August 2024. Trained CHWs screened all adults aged ≥ 40 years in intervention communities for hypertension, referring those with blood pressure (BP) ≥ 140/90 mmHg to MoH HIV/primary care clinics. After initial in‐clinic evaluation, adults with BP ≥ 160/100 mmHg were offered choice of clinic‐based or telehealth (CHW home visit, clinician telehealth evaluation, medication delivery) follow‐up care. Telehealth used a MoH‐compatible CHW smartphone app that syncs with electronic clinic records, prompts CHW follow‐up visits and facilitates clinician telehealth assessment/medication prescribing. We report hypertension control achieved through the implementation of CHW‐supported screening and telehealth and used targeted minimum loss‐based estimation to estimate the change in population prevalence of uncontrolled hypertension from baseline to 1 year.

**Results:**

Across eight communities, 198 CHWs measured BP in 14,378/15,879 adults aged ≥ 40 years at baseline (91%) and 13,334/15,879 after 1 year (84%); 55% were female and 19% living with HIV. Estimated population prevalence of BP ≥ 140/90 mmHg decreased from 16.0% at baseline to 6.4% at year 1 (9.6% absolute decrease, 95% CI 8.6%, 10.6%). Among people with HIV aged ≥ 40 years (*n* = 3036), the prevalence of BP ≥ 140/90 mmHg decreased from 10.5% to 4.7% (5.9% absolute decrease, 95% CI 3.0%, 8.8%). In the subset with BP ≥ 160/100 who enrolled in the intervention (*n* = 919), 96% received antihypertensive medication, 81% were retained in care at 1 year and 79% achieved BP control; people with HIV (*n* = 120) had similar retention (80%) and BP control (80%).

**Conclusions:**

Within the context of a pragmatic trial, leveraging existing CHWs in an integrated HIV/hypertension model reduced the population‐level prevalence of uncontrolled hypertension by 60% among people with and without HIV, extending health services into the community at scale.

## 1 INTRODUCTION

Hypertension is the leading modifiable cause of cardiovascular disease (CVD) globally and an increasingly common cause of morbidity and mortality in low‐ and middle‐income countries in Africa [1, 2]. In Kenya and Uganda, hypertension prevalence ranges from 13% to 27%, with higher prevalence observed among older adults, who face the greatest risk of CVD [3, 4, 5, 6]. Diagnosis, treatment and control are lower in Africa compared to other regions [7], including Kenya and Uganda, where fewer than one‐quarter of people with hypertension are diagnosed and fewer than 10% have controlled hypertension [5, 8, 9, 10].

People living with HIV are at increased risk of CVD due to a higher prevalence of traditional risk factors, as well as chronic inflammation associated with viral replication and exposure to antiretroviral therapy (ART) [11, 12, 13, 14, 15, 16, 17, 18, 19]. With the expansion of ART and the decline of AIDS‐associated morbidity and mortality, people living with HIV are living longer, and the burden of disease has shifted towards non‐communicable diseases and CVD in particular [11, 12]. The rising burden of CVD among people with HIV has prompted a frameshift: high‐quality HIV care can no longer be limited to ART delivery and prevention and treatment of opportunistic infections. CVD prevention must be included as part of routine HIV care—both to improve overall health and sustain gains made by the HIV response [20].

At the same time, the increasing burden of CVD across settings such as East Africa impacts both people with HIV and people without HIV. Care models must serve all people at risk of CVD to ensure effective, equitable and population‐level impact. Clinic‐based care models can provide HIV status‐neutral hypertension treatment, though are limited by either low levels of community reach, or by low retention in care. In the recent INTE‐AFRICA study, the integration of HIV, hypertension and diabetes treatment in Uganda and Tanzania resulted in high levels of retention in care over 1 year [21]. However, the INTE‐AFRICA study was conducted among people already engaged in care and thus was not designed to reach people with undiagnosed hypertension or those not currently in care. We previously showed in the SEARCH study that population‐level multi‐disease (HIV, hypertension, diabetes screening) community heath campaigns can improve hypertension diagnosis through community screening and improve hypertension control and all‐cause mortality through integrated patient‐centred clinic‐based care [4]. Despite these improvements, linkage to care and long‐term retention were sub‐optimal, with only 53% maintaining hypertension control after 3 years.

People with hypertension face multiple barriers to accessing hypertension screening and chronic hypertension care, including transportation challenges, competing priorities, medication availability and costs, and knowledge limitations [22]. These barriers are compounded when hypertension is severe or poorly controlled and requires frequent clinic visits to adjust medications and dosages. Community‐based approaches to hypertension diagnosis and treatment could help address many of these barriers to hypertension diagnosis and ongoing management.

Community health worker (CHW)‐facilitated hypertension screening outside of a clinic setting has been shown to improve hypertension diagnosis [23, 24, 25, 26, 27, 28]. The addition of CHW‐facilitated hypertension treatment could address multiple barriers to ongoing care engagement in hypertension care for both people with and without HIV. We previously conducted a pilot randomised trial of a CHW‐facilitated hypertension telehealth intervention where CHWs conducted home visits for blood pressure (BP) measurement, facilitated a telehealth call with a clinician and delivered clinician‐prescribed medications [29]. This intervention led to a nearly two‐fold improvement in hypertension control at 48 weeks compared to integrated clinic‐based care (86% vs. 44% hypertension control). We now seek to understand the effectiveness of this CHW telehealth intervention at scale. In the present study, we evaluated the implementation of HIV and hypertension CHW screening plus the CHW hypertension telehealth intervention at a community‐based population level in eight communities in Kenya and Uganda as part of an ongoing cluster randomised controlled trial.

## 2 METHODS

### 2.1 Study setting, design and participants

This prospective cohort study was nested within the intervention arm of an ongoing cluster‐randomised population‐level study in rural southwestern Uganda and western Kenya (SEARCH: NCT05768763). Intervention communities received a community‐based integrated HIV/hypertension intervention that included CHW hypertension screening, referral to clinic and CHW‐facilitated home‐based telehealth visits for those with moderate to severe hypertension. We evaluated changes in hypertension outcomes over 1 year among all adults aged ≥ 40 years residing in the eight intervention communities at baseline. Baseline hypertension screening began on 29 March 2023. The study is ongoing, and we report data through 27 August 2024 (the conclusion of year 1 follow‐up screening). The study received ethical approval from the Makerere University School of Medicine Research and Ethics Committee in Uganda (Mak‐SOMREC‐2023‐566); the Kenya Medical Research Institute in Kenya (P00151‐4173); and the University of California San Francisco in the United States of America (20‐32144).

### 2.2 Intervention and study procedures

Ministry of Health (MoH) CHWs received initial training on HIV counselling and testing, BP measurement, hypertension basics (overview of diagnostic criteria, medication names and purpose), and use of a CHW‐facing smartphone app to guide screening and subsequent hypertension treatment support with clinician telehealth evaluation. Following training, each CHW had 1:1 coaching for 1–5 days until proficient with intervention delivery. The integrated HIV/hypertension intervention consisted of two major components: (1) population‐level screening of all adults aged ≥ 15 years for HIV and aged ≥ 18 years for hypertension and (2) hypertension follow‐up home visits with clinician telehealth for adults aged ≥ 40 years with baseline moderate to severe hypertension (BP ≥ 160/100 mmHg). CHWs obtained verbal consent before baseline screening. Baseline screening included an offer of home‐based HIV testing to all adults not known to be living with HIV. People living with HIV who reported being off HIV treatment, were pregnant or who had a newly positive HIV antibody test were referred to the nearest government HIV clinic. For those with a negative HIV test, CHWs asked about self‐assessed HIV risk and referred those reporting risk to the nearest government primary health centre for enrolment in a nested Dynamic Choice HIV prevention intervention [30, 31, 32, 33, 34]. At household visits, CHWs conducted BP measurements using a standardised protocol, with a single BP measurement taken after 5 minutes of rest and two additional measures separated by at least 1 minute of rest for those with an initial BP ≥ 140 mmHg systolic or ≥ 90 mmHg diastolic [35]. Using the average of the second and third BP measure, everyone with BP ≥ 140/90 mmHg was referred to the nearest government primary health centre; those with BP ≥ 160/100 mmHg were given a transportation voucher redeemable for the equivalent of approximately $5 US Dollars upon linkage to hypertension care, based on our prior work [25]. CHWs called the study clinician for anyone with possible hypertensive urgency/emergency (defined as average BP ≥ 180/110 mmHg with possibly related symptoms or average BP ≥ 200/120 mmHg regardless of symptoms) for phone triage and arrangement of immediate referral to the clinic.

Upon linkage to the health facility, study clinicians repeated BP measurements using the same procedures. Participants were eligible for the embedded CHW‐facilitated hypertension telehealth intervention if BP was moderate‐severely elevated at community‐ or clinic‐based screening (≥ 160 mmHg systolic or ≥ 100 mmHg diastolic) and remained elevated to at least ≥ 140/90 mmHg at the time of clinic evaluation. Community residents (aged ≥ 40 years) who were screened at the clinic without prior CHW measurement were also eligible if their BP was ≥ 160 mmHg systolic or ≥ 100 mmHg diastolic. Participants with hypertensive urgency/emergency received immediate evaluation and treatment following country guidelines within the MoH clinic before the initiation of any study procedures. Participants completed written informed consent before enrolment. Hypertension treatment at the initial visit and all follow‐up visits was provided using a hypertension treatment algorithm developed based on country guidelines using locally available antihypertensive medications.

**Figure 1:**
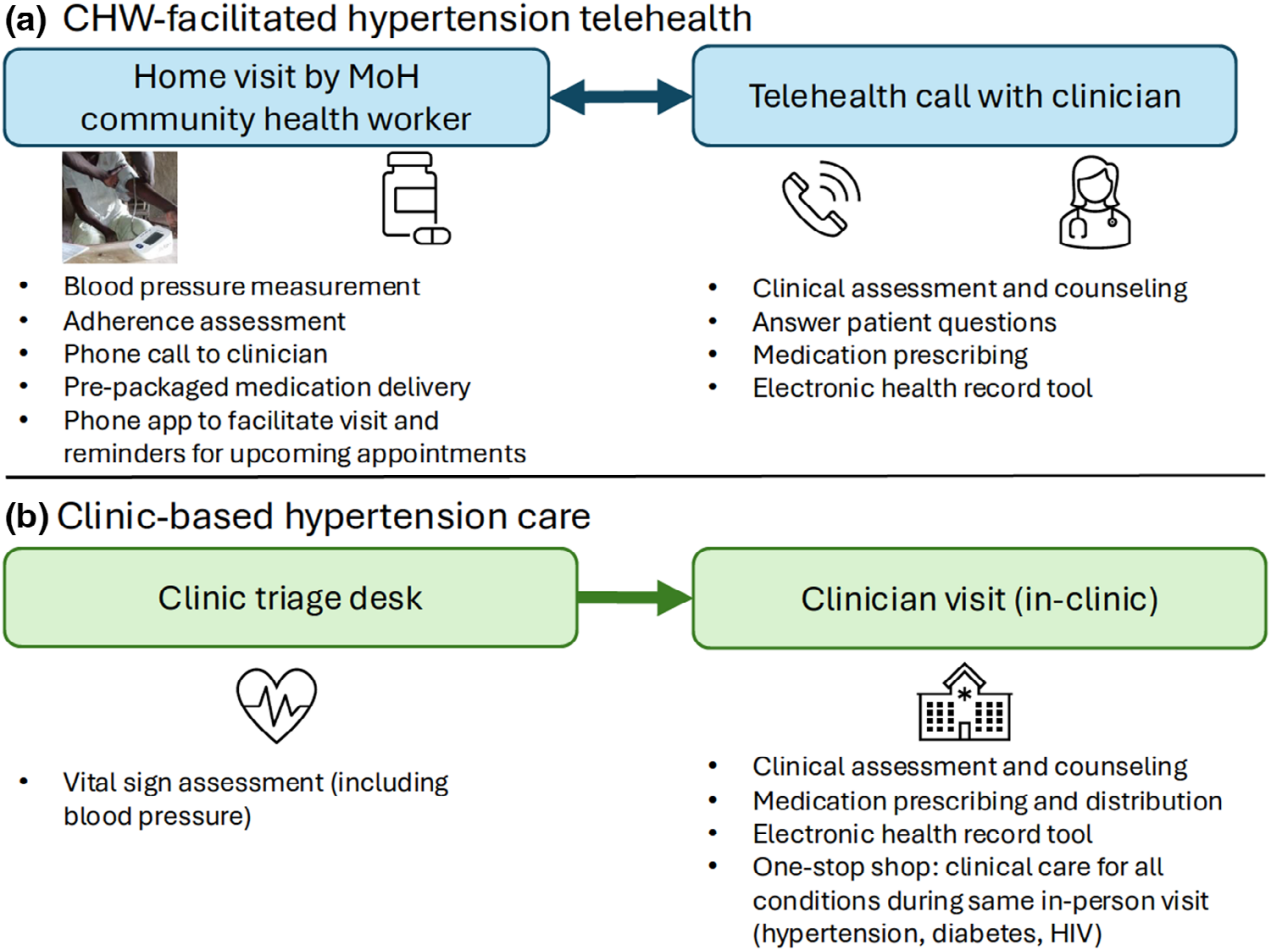
**Hypertension care models**. Participants were offered choice between CHW‐facilitated hypertension telehealth **(a)** or clinic‐based hypertension care **(b)**, with option for participants or clinicians to dynamically change choice over time based on preference or clinical need. In both care models, participants received free hypertension medications based on country‐specific treatment algorithms. Medication refill duration and follow‐up were scheduled every 4 weeks if blood pressure was uncontrolled (≥ 140/90 mmHg) or every 12 weeks if blood pressure was controlled (< 140/90 mmHg). Abbreviations: CHW, community health worker; MoH, Ministry of Health.

Following the initial visit, participants receiving the hypertension telehealth intervention were offered a choice of the next follow‐up location, either at the clinic or at home with a CHW facilitating clinician evaluation via telehealth as depicted in Figure 1. Table S1 provides additional details of both care models and, for reference, standard hypertension care that existed prior to the introduction of study interventions. Follow‐up visits were scheduled every 4 weeks if hypertension was uncontrolled and every 12 weeks if hypertension was controlled (defined as BP < 140/90 mmHg, based on country guidelines).

Hypertension telehealth visits consisted of a CHW home visit where CHWs assessed adherence, provided counselling on adherence and lifestyle changes to improve BP (e.g. salt reduction, physical activity), and measured BP using the standardised protocol described above. CHWs then called the study clinician who was stationed at the primary health centre in their community for a telehealth clinician visit. The clinician reviewed the data with the CHW, talked directly with the participant for clinical assessment and counselling, and prescribed medications which the CHWs delivered using pre‐packaged medication sets based on treatment algorithm steps.

CHWs used a smartphone app to enter all screening and follow‐up data. The app was built on the open‐source Medic Mobile platform [36] and was designed to walk CHWs through procedures for screening and telehealth follow‐up visits. The CHW app features two‐way synchronisation with the study electronic medical record available to clinic‐based clinicians: CHW‐entered data are available to clinicians, and the clinic database pushes upcoming appointment reminders and prompts for CHWs to conduct tracing for participants who missed hypertension, HIV or HIV prevention visits.

### 2.3 Measurements

CHWs entered baseline census data on community members residing in their assigned households and updated information on births, deaths and moves in/out of the community over the course of the study. One year following the initial screening, CHWs repeated population‐level HIV and hypertension screening among all adults residing in study communities using the same procedures described above. To assess HIV status, CHWs asked all residents about their HIV status at the time of screening. Those who reported negative or unknown status were offered rapid HIV testing, with positive or indeterminant tests leading to referral to the clinic for additional testing and, if diagnosed with HIV, linkage to care. We supplemented CHW assessment with linkage to the clinic medical record to identify people who were in HIV care but missed by community screening.

### 2.4 Analysis

We used targeted minimum loss‐based estimation (TMLE) to estimate the prevalence of uncontrolled hypertension (BP ≥ 140/90 mmHg) and moderate‐severe hypertension (BP ≥ 160/100 mmHg) at baseline and year 1 as well as evaluate changes over time. We accounted for differences between participants with and without BP measures. At baseline, we assumed that within all values of country, community, sex, age and HIV status, persons who screened at baseline were representative of persons who did not. We made a similar assumption at year 1, but expanded our adjustment set to include baseline screening. Within TMLE, we used Super Learner, an ensemble machine learning method, to minimise modelling assumptions and combine estimates from main terms regression, multivariate adaptive regression splines and the mean. We conducted several sensitivity analyses to examine the robustness of this approach (Table S5). We repeated these analyses within strata defined by HIV status (known vs. not known to have HIV), country and sex.

Among the subset who enrolled in the embedded CHW‐facilitated hypertension telehealth intervention, we describe participant characteristics and report care delivery, engagement, retention (defined as not late for a follow‐up visit by more than 7 days at the time of 1‐year population‐level screening) and achievement of hypertension control (defined as BP < 140/90 mmHg on average of three measures at any follow‐up visit for clinical hypertension care during the 1 year follow‐up period).

### 2.5 Role of the funding source

The funder of the study had no role in study design, data collection, data analysis, data interpretation or writing of the report.

## 3 RESULTS

### 3.1 Baseline screening

**Figure 2:**
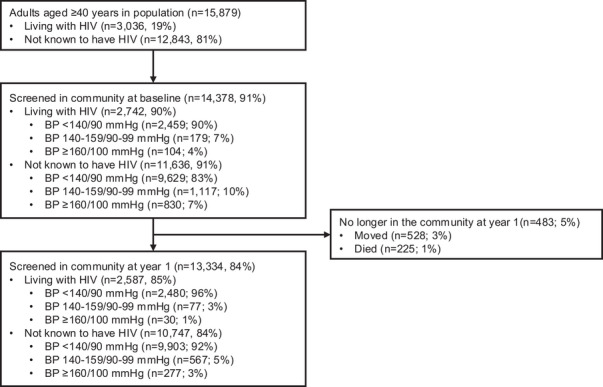
**Consort diagram for community health worker hypertension screening at baseline and year 1**. Abbreviations: BP, blood pressure; mmHg millimetres of mercury.

Of 42,121 adults aged ≥ 18 years in eight intervention communities, 15,879 were aged ≥ 40 years at baseline. Among adults aged ≥ 40 years, 198 CHWs measured BP in 14,378/15,879 (91%) at baseline (Figure 2). Among those screened, 55% were female and 19% were living with HIV (Table 1). Baseline BP measurement during community‐based screening was similar among people with HIV (90% screened for hypertension).

**Table 1:** Characteristics of participants at baseline

	Screened for hypertension at baseline (*n* = 14,378)	Not screened for hypertension at baseline (*n* = 1501)	Overall (*N* = 15,879)
**Age**			
Mean (SD)	55.5 (12.6)	53.4 (11.6)	55.3 (12.5)
Median [Q1, Q3]	52 [45, 63]	51 [44, 60]	52 [45, 63]
**Sex**			
Female	8084 (56.2%)	645 (43.0%)	8729 (55.0%)
Male	6294 (43.8%)	856 (57.0%)	7150 (45.0%)
**Country**			
Kenya	8098 (56.3%)	508 (33.8%)	8606 (54.2%)
Uganda	6280 (43.7%)	993 (66.2%)	7273 (45.8%)
**Living with HIV**	2742 (19.1%)	294 (19.6%)	3036 (19.1%)

*Note*: Characteristics of participants identified as members of the eight study communities during baseline census, stratified by completion of hypertension screening by community health worker at baseline.

Abbreviations: HIV, human immunodeficiency virus; SD, standard deviation.

### 3.2 Hypertension telehealth

**Figure 3:**
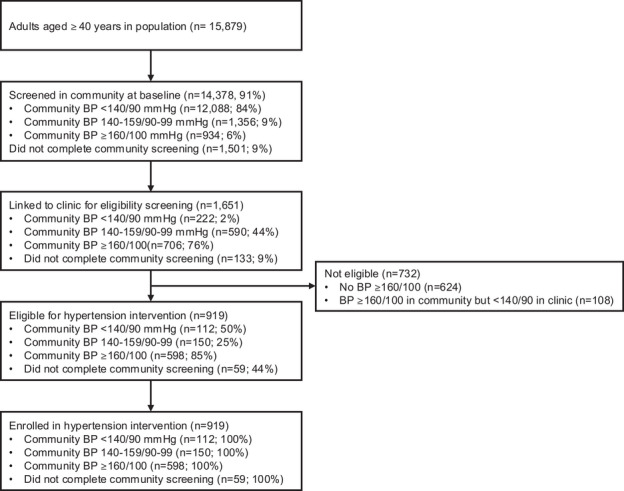
**Consort diagram for enrolment in CHW‐facilitated hypertension telehealth intervention**. *Note*: Participants could enrol in the CHW‐facilitated hypertension telehealth intervention if they had BP ≥ 160/100 mmHg on community screening and persistently elevated BP of at least ≥ 140/90 mmHg upon presentation to the clinic, or if they had BP ≥ 160/100 mmHg upon presentation to the clinic regardless of BP measurement in the community. Abbreviations: BP, blood pressure; CHW, community health worker.

Nine hundred and nineteen were eligible and enrolled in the CHW‐facilitated hypertension telehealth intervention: 598 were first identified with moderate‐severe hypertension (BP ≥ 160/100 mmHg) on community screening; 150 had mild hypertension (BP 140–159/90–99 mmHg) in the community and were identified with moderate‐severe hypertension upon clinic linkage, and 171 had normal or no community BP screening but were identified with moderate‐severe hypertension on clinic screening (Figure 3). Enrolled participants had a mean age of 62 years (SD 14 years); 66% were female sex, and 13% were living with HIV. At the initial clinic visit, 77% had BP ≥ 160/100 mmHg, and 19% had BP ≥ 180/110 mmHg (Table 2).

**Table 2:** Characteristics of participants in telehealth intervention

	Living with HIV (*n* = 115)	Without known HIV (*n* = 804)	Overall (*N* = 919)
**Age**			
Mean (SD)	55.8 (9.1)	62.9 (13.4)	62.0 (13.2)
Median [Q1, Q3]	54 [48, 62]	62 [51, 72]	61 [51, 71]
**Sex**			
Female	82 (71.3%)	528 (65.7%)	610 (66.4%)
Male	33 (28.7%)	276 (34.3%)	309 (33.6%)
**Country**			
Kenya	69 (60.0%)	355 (44.2%)	424 (46.1%)
Uganda	46 (40.0%)	449 (55.8%)	495 (53.9%)
**Living with HIV**	115 (100%)	−	115 (12.5%)
**SBP at enrolment**			
Mean (SD)	154.9 (14.9)	161.6 (16.7)	160.8 (16.6)
Median [Q1, Q3]	156 [142, 166]	160 [150, 172]	160 [150, 170]
**DBP at enrolment**			
Mean (SD)	100.7 (8.1)	99.3 (10.6)	99.5 (10.4)
Median [Q1, Q3]	100 [96, 106]	100 [93, 105]	100 [94, 105]
**BP at enrolment (mmHg)**			
140−159/90−99	27 (23.5%)	182 (22.6%)	209 (22.7%)
160−179/100−109	73 (63.5%)	466 (58.0%)	539 (58.7%)
≥ 180/110	15 (13.0%)	156 (19.4%)	171 (18.6%)

Abbreviations: BP, blood pressure; DBP, diastolic blood pressure; mmHg, millimetres of mercury; Q1, first quartile; Q3, third quartile; SBP, systolic blood pressure; SD, standard deviation.

Over the first year of implementation, 96% (*n* = 881/919) of participants who were enrolled in the CHW‐facilitated telehealth intervention received ≥ 1 antihypertensive medication; 94% (*n* = 860/919) had ≥ 1 follow‐up visit; 82% (*n* = 757/919) had at least one follow‐up visit by telehealth; 80% (*n* = 736/919) were retained in care at 1 year; and 79% (*n* = 730/919) achieved BP control during at least one follow‐up visit; people with HIV (*n* = 115) had a similar proportion who received ≥ 1 visit by telehealth (83%; *n* = 95/115), were retained in care (80%; *n* = 92/115) and achieved BP control (80%; *n* = 95/115) (Table 3 and Table S2).

**Table 3:** Hypertension intervention delivery, stratified by HIV status

	Living with HIV (*n* = 115)	Without known HIV (*n* = 804)	Overall (*N* = 919)
**Number of visits for hypertension care (including enrolment), median [Q1, Q3]**	5 [3, 7]	5 [4, 7]	5 [4, 7]
Telehealth visits, median [Q1, Q3]	3 [1, 5]	3 [1, 5]	3 [1, 5]
Clinic visits, median [Q1, Q3]	1 [1, 2]	1 [1, 2]	1 [1, 2]
**Attended at least one post‐enrolment follow‐up visit**	109 (94.8%)	751 (93.4%)	860 (93.6%)
Attended at least one post‐enrolment follow‐up visit by telehealth	95 (82.6%)	662 (82.3%)	757 (82.4%)
**Received ≥ 1 hypertension medication**	107 (93.0%)	774 (96.3%)	881 (95.9%)
**Achieved BP control < 140/90 mmHg during at least one follow‐up visit**	95 (82.6%)	635 (79.0%)	730 (79.4%)
**Retained in care at 1 year**	92 (80.0%)	644 (80.1%)	736 (80.1%)

Abbreviations: BP, blood pressure; mmHg, millimetres of mercury; Q1, first quartile; Q3, third quartile.

### 3.3 Year 1 screening

CHWs measured BP in 13,334/15,879 adults aged ≥ 40 after 1 year (84%). BP measurement completion at year 1 was similar among people living with HIV (85%; *n* = 2587/3306) (Figure 2 and Supporting information Tables S3 and S4).

### 3.4 Changes in population‐level hypertension over 1 year

**Figure 4:**
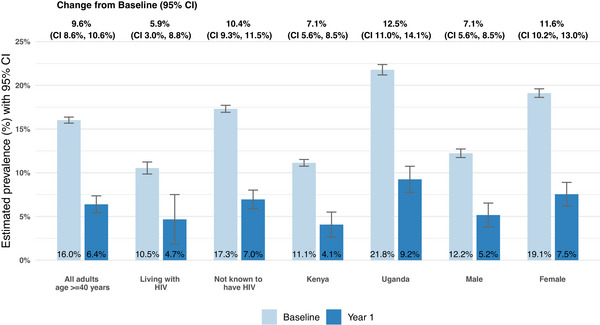
**Pre‐post comparison of population‐level uncontrolled hypertension from baseline to year 1**. *Note*: Estimated community‐wide, population‐level prevalence of uncontrolled hypertension (blood pressure ≥ 140/90 mmHg) at baseline and 1 year after introduction of intervention. Prevalence at both time points and change from baseline to 1 year estimated using targeted minimum loss‐based estimation (TMLE). Abbreviation: CI, confidence interval.

**Figure 5:**
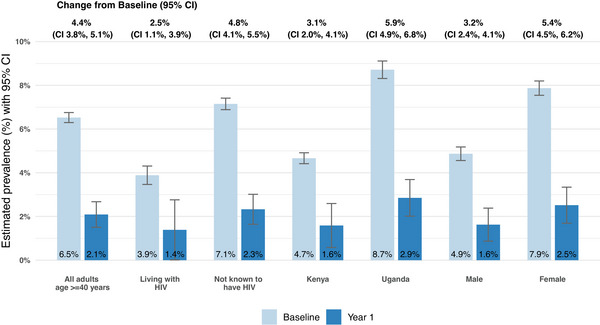
**Pre‐post comparison of population‐level uncontrolled moderate‐severe hypertension from baseline to year 1**. *Note*: Estimated community‐wide, population‐level prevalence of uncontrolled moderate‐severe hypertension (blood pressure ≥ 160/100 mmHg) at baseline and 1 year after introduction of intervention. Prevalence at both time points and change from baseline to 1 year estimated using targeted minimum loss‐based estimation (TMLE). Abbreviation: CI, confidence interval.

In pre‐post comparison, the estimated population‐level prevalence of BP ≥ 140/90 mmHg decreased from 16.0% at baseline (95% confidence interval [CI] 15.7%, 16.4%) to 6.4% at year 1 (95% CI 5.4%, 7.4%), a decrease of 9.6% (95% CI 8.6%, 10.6%). Estimated population‐level prevalence of BP ≥ 160/100 mmHg decreased from 6.5% at baseline (95% CI 6.3%, 6.8%) to 2.1% at year 1 (95% CI 1.5%, 2.7%), a 4.4% absolute decrease (95% CI 3.8%, 5.1%; Figures 4 and 5, and Table 4). These results were robust to the handling of missing data (Table S5).

**Table 4:** Pre‐post comparison of population‐level uncontrolled hypertension after 1 year

	Prevalence of BP ≥ 140/90			Prevalence of BP ≥ 160/90		
	Baseline	Year 1	Change	Baseline	Year 1	Change
**All adults aged ≥ 40 years**	16.0% (95% CI 15.7%, 16.4%)	6.4% (95% CI 5.4%, 7.4%)	9.6% (95% CI 8.6%, 10.6%)	6.5% (95% CI 6.3%, 6.8%)	2.1% (95% CI 1.5%, 2.7%)	4.4% (95% CI 3.8%, 5.1%)
**By HIV status**						
Living with HIV	10.5% (95% CI 9.9%, 11.2%)	4.7% (95% CI 1.8%, 7.5%)	5.9% (95% CI 3.0%, 8.8%)	3.9% (95% CI 3.5%, 4.3%)	1.4% (95% CI 0.0%, 2.8%)	2.5% (95% CI 1.1%, 3.9%)
Not known to have HIV	17.3% (95% CI 16.9%, 17.7%)	7.0% (95% CI 5.9%, 8.0%)	10.4% (95% CI 9.3%, 11.5%)	7.1% (95% CI 6.9%, 7.4%)	2.3% (95% CI 1.6%, 3.0%)	4.8% (95% CI 4.1%, 5.5%)
**By country**						
Kenya	11.1% (95% CI 10.8%, 11.5%)	4.1% (95% CI 2.6%, 5.5%)	7.1% (95% CI 5.6%, 8.5%)	4.7% (95% CI 4.4%, 4.9%)	1.6% (95% CI 0.6%, 2.6%)	3.1% (95% CI 2.0%, 4.1%)
Uganda	21.8% (95% CI 21.2%, 22.4%)	9.2% (95% CI 7.7%, 10.7%)	12.5% (95% CI 11.0%, 14.1%)	8.7% (95% CI 8.3%, 9.1%)	2.9% (95% CI 2.0%, 3.7%)	5.9% (95% CI 4.9%, 6.8%)
**By sex**						
Male	12.2% (95% CI 11.7%, 12.7%)	5.2% (95% CI 3.8%, 6.5%)	7.1% (95% CI 5.6%, 8.5%)	4.9% (95% CI 4.6%, 5.2%)	1.6% (95% CI 0.9%, 2.4%)	3.2% (95% CI 2.4%, 4.1%)
Female	19.1% (95% CI 18.6%, 19.6%)	7.5% (95% CI 6.2%, 8.9%)	11.6% (95% CI 10.2%, 13.0%)	7.9% (95% CI 7.5%, 8.2%)	2.5% (95% CI 1.7%, 3.3%)	5.4% (95% CI 4.5%, 6.2%)

*Note*: Estimated prevalence of uncontrolled hypertension (BP ≥ 140/90 mmHg) and uncontrolled moderate‐severe hypertension (BP ≥ 160/100 mmHg) using targeted minimum loss‐based estimation with adjustment for missing blood pressure measurements.

Abbreviations: BP, blood pressure; CI, confidence interval.

Among people with HIV aged ≥ 40 years (*n* = 3306), estimated prevalence of BP ≥ 140/90 mmHg decreased from 10.5% at baseline (95% CI 9.9%, 11.2%) to 4.7% at year 1 (95% CI 1.8%, 7.5%), a decline of 5.9% (95% CI 3.0%, 8.8%). The prevalence of BP ≥ 160/100 mmHg decreased from 3.9% at baseline (95% CI 3.5%, 4.3%) to 1.4% at year 1 (95% CI 0%, 2.8%), a 2.5% absolute decrease (95% CI 1.1%, 3.9%).

Baseline prevalence of uncontrolled hypertension was greater in Uganda (21.8%; 95% CI 21.2%, 22.4%) compared to Kenya (11.1%; 95% CI 10.8%, 11.5%), among people not known to have HIV (17.3%, 95% CI 16.9%, 17.7%) compared to people living with HIV (10.5%, 95% CI 9.9%, 11.2%) and among females (19.1%, 95% CI 18.6%, 19.6%) compared to males (12.2%, 95% CI 11.7%, 12.7%). Despite baseline differences, there were similar relative declines in prevalence of uncontrolled hypertension over the 1‐year intervention across all sub‐groups.

## 4 DISCUSSION

In this population‐level study, we showed that combined CHW multi‐disease screening and CHW‐facilitated hypertension telehealth intervention led to a 60% reduction in the population prevalence of uncontrolled hypertension over 1 year (from 16.0% to 6.4%) among adults aged ≥ 40 years across eight rural communities in Kenya and Uganda. We observed reductions in population‐level uncontrolled hypertension across all sub‐groups, including among people with HIV.

We also demonstrated that MoH CHWs can achieve a high level of hypertension screening coverage through a multi‐disease HIV, HIV risk and hypertension screening package. Other studies have shown that CHWs can successfully conduct community‐based hypertension screening; we add to this literature by demonstrating a high uptake of hypertension screening at scale across multiple communities in rural East Africa [23, 24, 25, 26, 27, 28].

Despite high levels of HIV care engagement among people with HIV, with Kenya exceeding UNAIDS 95‐95‐95 targets and Uganda nearing 90‐90‐90 targets [37], we identified a high prevalence of uncontrolled hypertension at baseline among people living with HIV suggesting missed opportunities for hypertension diagnosis and treatment during routine HIV care. People living with HIV experienced large reductions in uncontrolled hypertension by year 1, suggesting that our CHW screening and facilitated telehealth intervention successfully extended traditionally clinic‐based services to the community for people living with HIV. As efforts expand to integrate hypertension and other non‐communicable disease services into HIV clinics, CHW‐facilitated telehealth provides an alternative that reduces the need for all services to be delivered within a single integrated visit. Further, telehealth avoids requiring people to come to the clinic for frequent visits for hypertension medication titration that occur more frequently than visits for stable HIV care which may be only twice yearly.

Among people with moderate to severe baseline hypertension regardless of HIV status, our CHW‐facilitated hypertension telehealth intervention resulted in high levels of retention in care and hypertension control over the 1‐year study period. People with moderate to severe hypertension are at greatest risk of near‐term morbidity and mortality from CVD and stand to benefit most from achieving hypertension control [38, 39, 40]. We previously showed that population‐level hypertension screening and clinic‐based integrated care reduced all‐cause mortality among people with hypertension in rural Kenya and Uganda, with the greatest benefits among those with moderate to severe hypertension [4]. In our present study, CHW‐facilitated telehealth resulted in greater retention in care than prior clinic‐based care models and has the potential to further reduce CVD morbidity and mortality in this high‐risk population.

Our study adds to a growing body of literature showing the effectiveness of CHW‐based interventions for hypertension treatment outside of a traditional clinic setting. Two trials in middle‐income countries (Colombia, Malaysia, Argentina) showed that home‐based hypertension treatment that was CHW‐led and primary care physician supervised improved BP control compared to standard clinic‐based care (achieving 69–73% hypertension control in home‐based CHW intervention arms compared to 30–52% in clinic‐based care) [41, 42]. Another small study in Uganda showed that in‐person visits by a clinician and a CHW in the community improved access to care and reduced BP more effectively than district hospital‐based care [43]. Our study builds on this literature by incorporating primary care clinician supervision and clinical evaluation by telehealth into CHW home visits at a population level. Our CHW‐facilitated, clinician‐supervised telehealth model has the potential for scalability across different settings where CHWs are present, including Kenya and Uganda. A future study is planned to understand the implementation strategies needed to facilitate embedding and sustaining this care model within routine care and cost and cost‐effectiveness of this approach.

Our study has several limitations. First, our single‐arm analysis lacks a contemporary comparison group, so we cannot rule out temporal trends as the explanation for our observed reductions in population‐level hypertension prevalence. However, it is unlikely that such large reductions would occur due to temporal changes, given the slow uptake of hypertension treatment in primary health centres in our setting to date. Following the completion of the cluster randomised trial, we will be able to compare population‐level hypertension outcomes between trial arms. Second, our study was conducted within a controlled research environment with study clinicians and financing for CHW stipends. While this provides evidence for effectiveness at scale, further studies are needed to understand implementation strategies to facilitate implementation of CHW‐facilitated hypertension telehealth within routine MoH primary health centres. Third, as a large population‐level study, we have limited individual measures of patient experience with the CHW‐facilitated hypertension telehealth model. We previously reported additional information on potential mechanisms for how CHW‐facilitated hypertension telehealth likely functions to improve outcomes in the pilot study that informed the larger trial [29] and will also further explore mechanisms through nested qualitative studies. Fourth, although we achieved high coverage of BP measurements at both baseline and year 1, we were unable to measure BP in everyone. While this high coverage limits the impact of missing data on our estimates, we further adjusted for differences between those with and without measurements using TMLE. Our results were robust to this approach and several sensitivity analyses, strengthening our inferences.

## 5 CONCLUSIONS

Population‐level uncontrolled hypertension was reduced over the first year of a CHW multi‐disease screening and facilitated hypertension telehealth intervention in Kenya and Uganda. By extending care to the community, this approach may be an effective strategy for improving hypertension outcomes and preventing CVD for both People living with and without HIV.

## COMPETING INTERESTS

The authors have declared that no competing interests exist.

## AUTHORS’ CONTRIBUTIONS

MDH: Initial drafting of the manuscript. MDH, EWM, JS and LBB: Data management and analysis. MDH, AO, SO, HS, CA, NS, GA, JK, EK, NS, DB, AM, GC, DVH, MRK and MLP: Study management and data collection.

DVH, MLP and MRK: Acquisition of funding and study oversight. All authors reviewed and approved the final manuscript.

## FUNDING

The study was supported by grants from the National Institutes of Health (U01‐AI150510 Multiple‐PI award to DVH, MLP, MRK; K23HL162578 to MDH).

## PATIENT CONSENT STATEMENT

Participants provided verbal consent prior to the completion of screening. Participants eligible for the severe hypertension telehealth intervention provided written informed consent prior to enrolment.

## CLINICAL TRIAL REGISTRATION

The study is registered on clinicaltrials.gov (NCT05768763).

## Supporting information




**Table S1**: Characteristics of hypertension care under standard and intervention conditions.
**Table S2**: Hypertension intervention delivery, stratified by sex.
**Table S3**: Characteristics of participants with and without blood pressure measurement at year 1.
**Table S4**: Community health worker blood pressure measurement and unadjusted prevalence of uncontrolled hypertension at baseline and year 1.
**Table S5**. Sensitivity analyses and results.

## Data Availability

A complete de‐identified patient dataset sufficient to reproduce the study findings will be made available approximately 1 year after completion of the ongoing trial (NCT04810650), following approval of a concept sheet summarising the analyses to be performed. Further inquiries can be directed to the SEARCH Scientific Committee at douglas.black@ucsf.edu
