# Supplementary material for: A prospective cohort study of the SEARCH integrated HIV/hypertension community health worker‐led intervention in rural Kenya and Uganda
Source: J Int AIDS Soc. 2025 Jul 7;28(Suppl 3):e26500. doi: 10.1002/jia2.26500 (PMC12232486; doi:10.1002/jia2.26500)
Supplement: Supplementary file 1 — Table S1: Characteristics of hypertension care under standard and intervention conditions. Table S2: Hypertension intervention delivery, stratified by sex. Table S3: Characteristics of participants with and without blood pressure measurement at year 1. Table S4: Community health worker blood pressure measurement and unadjusted prevalence of uncontrolled hypertension at baseline and year 1. Table S5. Sensitivity analyses and results. [file JIA2-28-e26500-s001.docx]

Supplemental materials

[Table S1. Characteristics of hypertension care under standard and intervention conditions 2](#_Toc193430710)

[Table S2. Hypertension intervention delivery, stratified by sex 4](#_Toc193430711)

[Table S3. Characteristics of participants with and without blood pressure measurement at year 1 5](#_Toc193430712)

[Table S4. Community health worker blood pressure measurement and unadjusted prevalence of uncontrolled hypertension at baseline and year 1 6](#_Toc193430713)

[Table S5. Sensitivity analyses and results 7](#_Toc193430714)

## Table S1. Characteristics of hypertension care under standard and intervention conditions

|  | **Standard of care**  **(prior to intervention)** | **Intervention option A – Home visits with telehealth*** | **Intervention option B – integrated care at clinic*** |
| --- | --- | --- | --- |
| **WHEN** | Generally monthly | Monthly if blood pressure uncontrolled  Every 3 months if blood pressure controlled | |
| **WHERE** | Primary care clinic or district hospital specialty clinic   - Hypertension care delivered in separate hypertension clinic or outpatient department (not integrated with HIV care) | Home  Clinician assessment over telehealth | Primary care clinic   - Integrated care for HIV and hypertension during same visit (if both conditions present) |
| **WHO** | Physician or  clinical officer (mild-level provider) | CHW  Clinical officer | Triage nurse  Clinical officer |
| **WHAT – provider actions** | - Blood pressure measurement - Clinical assessment and counselling - Medication prescribing | CHW:   - Blood pressure measurement - Adherence assessment - Phone call to clinician - Delivery of medication prescribed by clinician (1-3 months; pre-packed and delivered during visit in most cases) - Use of smartphone app to guide visit and provide reminders for upcoming visits   Clinical officer:   - Symptom assessment - Adherence counselling - Answer patient questions - Medication prescribing | - Blood pressure measurement at clinic triage - Clinical assessment and counselling - Medication prescribing |
| **WHAT – clinic-level factors/ interventions** | - Medication stock-outs common - Medications purchased for a fee - Paper-based hypertension registry | - Medication supply strengthening to avoid stock-outs - Medications provided free of charge - Electronic health record to record and monitor longitudinal hypertension data - Smartphone app for CHWs to guide visit and provide reminders for upcoming visits - Provider and CHW training on delivering patient-cantered, friendly services and advocating to minimize patient barriers to care - Introduction of simplified hypertension treatment algorithm with discrete treatment steps to facilitate pre-packaging of medications | |

* participants could choose their preferred location of hypertension follow-up care – either home-based with telehealth or clinic-based. Choice was dynamic and could change over time between these locations. Abbreviations: CHW, community health worker.

## Table S2. Hypertension intervention delivery, stratified by sex

|  | **Female (N=610)** | **Male (N=309)** | **Overall (N=919)** |
| --- | --- | --- | --- |
| **Number of visits for hypertension care (including enrollment), median [Q1, Q3]** | 5 [4, 7] | 5 [3, 6] | 5 [4, 7] |
| Telehealth visits, median [Q1, Q3] | 4 [2, 5] | 2 [1, 4] | 3 [1, 5] |
| Clinic visits, median [Q1, Q3] | 1 [1, 2] | 1 [1, 3] | 1 [1, 2] |
| **Attended at least one post-enrollment follow-up visit** | 575 (94.3%) | 285 (92.2%) | 860 (93.6%) |
| Attended at least one post-enrollment follow-up visit by telehealth | 513 (84.1%) | 244 (79.0%) | 757 (82.4%) |
| **Received ≥1 hypertension medication** | 590 (96.7%) | 291 (94.2%) | 881 (95.9%) |
| **Achieved BP control <140/90 mmHg during at least one follow-up visit** | 487 (79.8%) | 243 (78.6%) | 730 (79.4%) |
| **Retained in care at 1 year** | 502 (82.3%) | 234 (75.7%) | 736 (80.1%) |

Abbreviations: Q1, first quartile; Q3, third quartile; BP, blood pressure; mmHg, millimeters of mercury

## Table S3. Characteristics of participants with and without blood pressure measurement at year 1

|  | **Screened for hypertension at year 1 (N=13,334)** | **Not screened for hypertension at year 1 (N=2,545)** | **Overall (N=15,879)** |
| --- | --- | --- | --- |
| **Age** |  |  |  |
| Mean (SD) | 55.3 (12.4) | 54.9 (13.3) | 55.3 (12.5) |
| Median [Q1, Q3] | 52 [45, 63] | 51 [44, 62] | 52 [45, 63] |
| **Sex** |  |  |  |
| Female | 7,539 (56.5%) | 1,190 (46.8%) | 8,729 (55.0%) |
| Male | 5,795 (43.5%) | 1,355 (53.2%) | 7,150 (45.0%) |
| **Country** |  |  |  |
| Kenya | 7,630 (57.2%) | 976 (38.3%) | 8,606 (54.2%) |
| Uganda | 5,704 (42.8%) | 1,569 (61.7%) | 7,273 (45.8%) |
| **Living with HIV** | 2,587 (19.4%) | 449 (17.6%) | 3,036 (19.1%) |
| **Blood pressure measured at baseline screening** | 12,629 (94.7%) | 1,749 (68.7%) | 14,378 (90.5%) |
| **Baseline BP, among measured (mmHg)** |  |  |  |
| <140/90 | 10,706 (84.8%) | 1,382 (79.0%) | 12,088 (84.1%) |
| 140-159/90-99 | 1,163 (9.2%) | 193 (11.0%) | 1,356 (9.4%) |
| ≥160/100 | 760 (6.0%) | 174 (9.9%) | 934 (6.5%) |

Characteristics of participants with and without measured blood pressure during community health worker screening at year 1. Abbreviations: SD, standard deviation, HIV, human immunodeficiency virus, BP, blood pressure; mmHg millimeters of mercury.

## Table S4. Community health worker blood pressure measurement and unadjusted prevalence of uncontrolled hypertension at baseline and year 1

|  | **Living with HIV** | **Not known to have HIV** | **Overall** |
| --- | --- | --- | --- |
| BP measured at baseline | 90% (n=2,742/3,036) | 91% (n=11,636/12,843) | 91% (n=14,378/15,879) |
| BP at baseline (mmHg) |  |  |  |
| <140/90 | 90% (n=2,459/2,742) | 83% (n=9,629/11,636) | 84% (n=12,088/14,378) |
| 140-159/90-99 | 7% (n=179/2,742) | 10% (n=1,117/11,636) | 9% (n=1,356/14,378) |
| ≥160/100 | 4% (n=104/2,742) | 7% (n=830/11,636) | 6% (n=934/14,378) |
| BP measured at year 1 | 85% (n=2,587/3,036) | 84% (n=10,747/12,843) | 84% (n=13,334/14,378) |
| BP at year 1 (mmHg) |  |  |  |
| <140/90 | 96% (n=2,480/2,742) | 92% (n=9,903/11,636) | 93% (n=12,383/14,378) |
| 140-159/90-99 | 3% (n=77/2,742) | 5% (n=567/11,636) | 5% (n=644/14,378) |
| ≥160/100 | 1% (n=30/2,742) | 3% (n=277/11,636) | 2% (n=307/14,378) |

Abbreviations: BP, blood pressure; mmHg, millimeters of mercury

## Table S5. Sensitivity analyses and results

Our primary analysis used targeted minimum loss-based estimation (TMLE) to flexibly and robustly estimate the outcome prevalences at baseline and follow-up year 1 as well as evaluate changes over time. This approach relied on the following missing data assumption: at a given timepoint and after adjustment for demographics, HIV status and prior screening, participants whose blood pressure was measured were representative of participants whose blood pressure was not measured. This approach uses data on all participants, including those who had missing blood pressure at baseline or follow-up year 1. This approach also use machine learning to minimize modeling assumptions.

We conducted the following sensitivity analyses to examine the robustness of our results:

- “Exclude censored”: For follow-up year 1 estimates, exclude (versus censor and adjust) participants who moved or died during follow-up. For both timepoints, use TMLE to adjust for differences between the remaining participants with and without blood pressure measures.
- “Exclude missing”: For baseline and follow-up year 1 estimates, exclude participants without blood pressure measures at the correspond timepoint. Among the remaining participants, estimate prevalence and changes with the unadjusted estimator (i.e., the raw proportion among measured). This approach corresponds to the missing completely at random (MCAR) assumption.
- “Impute”: For follow-up year 1 estimates, impute missing blood pressure measures with baseline measures (if available). For both timepoints, use TMLE to adjust for differences between the remaining participants with and without blood pressure measures. This is the most conservative approach to missing data because it assumes no effect of the hypertension intervention among those with missing year 1 blood pressure values.

As shown in the following Table, our results were quite robust to analytic approach. This is not surprising given the high level of blood pressure coverage at both timepoints.

|  | **Approach** | **Baseline** | **Year 1** | **Change** |
| --- | --- | --- | --- | --- |
| Uncontrolled HTN | Primary | 16.0% (15.7,16.4%) | 6.4% (5.4,7.4%) | 9.6% (8.6,10.6%); p<0.001 |
|  | Exclude censored | 16.0% (15.7,16.4%) | 6.4% (5.4,7.5%) | 9.6% (8.5,10.7%); p<0.001 |
|  | Exclude missing | 15.9% (15.3,16.5%) | 7.1% (6.7,7.6%) | 8.8% (8.2,9.4%); p<0.001 |
|  | Impute | 16.0% (15.4,16.6%) | 8.8% (8.3,9.2%) | 7.2% (6.7,7.8%); p<0.001 |
| Severe HTN | Primary | 6.5% (6.3,6.8%) | 2.1% (1.5,2.7%) | 4.4% (3.8,5.1%); p<0.001 |
|  | Exclude censored | 6.5% (6.3,6.8%) | 2.1% (1.5,2.8%) | 4.4% (3.7,5.1%); p<0.001 |
|  | Exclude missing | 6.5% (6.1,6.9%) | 2.3% (2.0,2.6%) | 4.2% (3.7,4.6%); p<0.001 |
|  | Impute | 6.5% (6.1,6.9%) | 3.2% (2.9,3.5%) | 3.3% (2.9,3.7%); p<0.001 |
